# A National Retrospective Cohort Study Comparing the Effects of Cefepime Versus Piperacillin-Tazobactam on the Development of Severe Acute Kidney Injury in Patients With Septic Shock

**DOI:** 10.1093/cid/ciae600

**Published:** 2024-12-05

**Authors:** Asad E Patanwala, David E Nix, Thomas E Hills, Brian L Erstad

**Affiliations:** Faculty of Medicine and Health, School of Pharmacy, The University of Sydney, Sydney, New South Wales, Australia; Department of Pharmacy, Royal Prince Alfred Hospital, Camperdown, New South Wales, Australia; Department of Pharmacy Practice and Science, College of Pharmacy, University of Arizona, Tucson, Arizona, USA; Medical Research Institute of New Zealand, Wellington, New Zealand; Department of Infectious Diseases, Auckland City Hospital, Auckland, New Zealand; Department of Pharmacy Practice and Science, College of Pharmacy, University of Arizona, Tucson, Arizona, USA

**Keywords:** sepsis, septic shock, β-lactams, acute kidney injury, mortality

## Abstract

**Background:**

Cefepime and piperacillin-tazobactam are commonly used broad-spectrum antibiotics used to treat patients with potential gram-negative bacterial sepsis. Piperacillin-tazobactam has been shown to be associated with acute kidney injury (AKI). However, it has not been compared with cefepime in patients with septic shock. We compared the effects of cefepime and piperacillin-tazobactam on the incidence of severe AKI in patients with septic shock.

**Methods:**

This was a retrospective, multicenter, inverse probability-of-treatment weighted cohort study conducted in 220 geographically diverse community and teaching hospitals across the United States. Adult patients were included if they had septic shock on hospital admission and received cefepime or piperacillin-tazobactam. The proportions of patients in whom stage 3 AKI occurred during hospitalization were compared between groups.

**Results:**

Of the 8427 patients included in the final cohort, 4569 received cefepime and 3858 received piperacillin-tazobactam. Patients had a mean (SD) age of 66.2 (15.2) years, and 45.3% were female; the mean (SD) estimated glomerular filtration rate was 48 (24) mL/min/1.73 m^2^ on the day of admission. In the weighted cohort, stage 3 AKI occurred in 9.9% receiving cefepime and 9.8% receiving piperacillin-tazobactam (odds ratio, 0.98 [95% confidence interval, .84–1.15]; *P* = .82). In terms of secondary outcomes, there was no significant difference between cefepime and piperacillin-tazobactam with regard to renal replacement therapy, in-hospital death, major adverse kidney events, stage 1 AKI, stage 2 AKI, maximum recorded serum creatinine, or hospital length of stay

**Conclusions:**

Among hospitalized patients with septic shock, there was no difference between cefepime and piperacillin-tazobactam in the occurrence of severe AKI.

## Teaser

In this study of patients hospitalized for septic shock, conducted in 220 geographically diverse community and teaching hospitals across the United States, there was no difference between cefepime and piperacillin-tazobactam in the occurrence of acute kidney injury.

International practice guidelines formulated by the Surviving Sepsis Campaign and published in 2021 recommend that adults with sepsis or septic shock at low risk for multiple drug-resistant organisms receive a single antimicrobial agent with gram-negative bacterial coverage, albeit based on a very low quality of evidence [1]. This usually includes the use of a antipseudomonal β-lactam. The choice of antipseudomonal β-lactam for broad-spectrum gram-negative coverage varies by provider and institution, but the most prescribed agents include cefepime and piperacillin-tazobactam [2].

Observational studies involving diverse populations of hospitalized patients with infections of varying etiologies suggest possible differences in the adverse effect profiles of these 2 agents, particularly with respect to a higher incidence of acute kidney injury (AKI) with piperacillin-tazobactam, although the findings between studies were inconsistent [3–5]. The largest randomized controlled trial that compared cefepime with piperacillin-tazobactam in 2511 adults at a single academic medical center in the United States found no difference between groups for the primary outcome of the highest stage of AKI or death by day 14 [2]. Similarly, for secondary outcomes there was no significant difference between groups for major adverse kidney events (MAKEs) at 14 days, although therapy with cefepime resulted in a higher incidence of neurological dysfunction, as noted by fewer days alive and free of delirium and coma within 14 days (odds ratio [OR], 0.79 [95% confidence interval (CI), .65–.95]). It is important to note that 77.6% received concomitant vancomycin [2]. Approximately 54% of patients in each group had sepsis at baseline, as defined by Sepsis-3 criteria, and only 13% had septic shock [2].

No large study to our knowledge has compared cefepime to piperacillin-tazobactam in patients with septic shock for clinically important outcomes such as severe AKI. Patients in septic shock may be more vulnerable to developing AKI, and the difference in nephrotoxicity between these antibiotics may be more apparent. Therefore, the purpose of this multicenter retrospective cohort study was to determine if there is a difference between cefepime and piperacillin-tazobactam in the outcome of severe AKI in patients with septic shock.

## METHODS

### Study Design and Setting

This was a retrospective, multicenter, cohort study that included 220 hospitals across the United States. The PINC AI Healthcare Database was used, which is an electronic US hospital-based service level, all-payer database containing information on inpatient admissions, from geographically diverse community and teaching hospitals [6]. The Strengthening the Reporting of Observational Studies in Epidemiology (STROBE) guidelines were followed for all aspects of the study [7].

### Patients

We included patients admitted with septic shock between 1 September 2020 and 30 June 2022. Identification of septic shock was based on *International Classification of Diseases, Tenth Revision (ICD-10*) code R65.21 (severe sepsis with septic shock) present on admission and treated with intravenous vasopressors (norepinephrine, epinephrine, dopamine, vasopressin, or angiotensin II) on the day of admission. Inpatient encounters categorized as emergency or urgent admissions were selected. Adult patients (aged ≥18 years) were included who received cefepime or piperacillin-tazobactam in the hospital with the first dose on the day of hospital admission. This was done to emulate a clinical trial where recruitment would occur soon after admission. There were no restrictions for inclusion based on dose or duration of therapy. We excluded patients who were started on renal replacement therapy (RRT) on the day of admission, had a history of end-stage renal disease (*ICD-10* N18.6), had an estimated glomerular filtration rate (eGFR) <15 mL/min/1.73 m^2^, or had stage 3 AKI on the day on admission. These latter patients with stage 3 AKI were considered to already have reached the primary outcome.

### Baseline Variables

Baseline variables were collected on the day of admission. These included demographics (age, sex, race/ethnicity), hospital characteristics (urban vs rural, teaching vs nonteaching, and region), invasive mechanical ventilation, Charlson Comorbidity Index (CCI), renal disease (from the CCI), moderate-severe liver disease (from the CCI), infection source, body mass index, Acute Physiology and Chronic Health Evaluation (APACHE) II score, and pertinent laboratory measures (eGFR, serum creatinine [SCr], serum lactate, and serum chloride). The eGFR was obtained as reported in each hospital system and not derived. We also included specific antibiotics as baseline variables that were administered before the primary outcome. These were vancomycin and gram-negative broad-spectrum agents.

### Comparator Groups

We compared patients who received cefepime or piperacillin-tazobactam, with the first dose on day of admission. Similar to a clinical trial, the intervention was selected to be administered on admission, which is early during hospitalization.

### Outcomes

The primary outcome was stage 3 AKI, according to the Kidney Disease: Improving Global Outcomes (KDIGO) definition, based on the SCr component [8] (Supplementary Table 1). Secondary outcomes were the RRT, in-hospital mortality, in-hospital MAKE (stage 3 AKI at time of hospital discharge, death, or new dialysis) [9], stage 1 AKI, stage 2 AKI, maximum SCr, and hospital length of stay. As eGFR and SCr values on the day of admission may not represent the normal baseline before admission, the baseline eGFR for MAKEs was back-calculated, as recommended by KDIGO [8] and similarly to a major clinical trial [2].

### Statistical Analysis

Propensity scores were calculated using logistic regression, defined as the conditional probability of being treated with piperacillin-tazobactam. All baseline variables were included in the model. The propensity scores were used to derive inverse probability of treatment weights (IPTWs). The standardized mean difference (SMD) was reported before and after IPTW weighting in a love plot. An SMD of <0.1 for each baseline variable was indicative of balanced groups [10]. There were missing data for body mass index (29.5%) and a few laboratory parameters, including lactate (21.0%) and chloride (3.2%). These were imputed before weighting using multiple imputation (20 imputed data sets). Baseline continuous variables were reported as mean (SD) or median (interquartile range [IQR]) based on the normality of their distributions, which was ascertained visually.

The association between cefepime or piperacillin-tazobactam and the primary and secondary outcomes was determined using IPTW-weighted logistic or linear regression models, as appropriate. The ORs with 95% CIs were reported for the primary analysis. IPTW analyses were conducted using the survey [11] and MatchThem [12] packages, which return standard errors robust to the lack of pseudo–population independence in IPTW models. Two-sided *P* values <.05 were considered to indicate statistically significant for all analyses. Statistical analysis was conducted using R software (version 4.0.3; R Foundation for Statistical Computing).

Three sensitivity analyses were conducted. First, we accounted for clustering by hospital by conducting an IPTW-weighted mixed effects model with hospital as a random effect. Second, we conducted Fine-Gray competing risk regression model for the subdistribution hazard ratio of stage 3 AKI with death as a competing risk. Third, we compared groups when the index antibiotic was used for ≥2 days (days 1 and 2).

Prespecified subgroup analyses were also conducted based on selected baseline variables. The heterogeneity of treatment effect was based on tests of interaction in each model. As 8 tests were performed, the family-wise error rate was 0.38 (ie, 38% probability of ≥1 type 1 errors). Subgroups included age (<65 vs ≥65 years), sex (male vs female), invasive mechanical ventilation (yes vs no), obese status (obese vs nonobese), renal disease before admission, eGFR (<60 vs ≥60 mL/min/1.73 m^2^), vancomycin use (yes vs no), and use of gram-negative broad-spectrum antibiotic use (other than the index antibiotic; yes vs no). IPTW was recalculated for each subgroup for analysis. The ORs with 95% CIs were depicted in each subgroup via a forest plot.

### Ethical Considerations

The study was approved by the executive of the Ethics Review Committee of the Sydney Local Health District (April 2024), who determined that ethical review by the committee was not required as the data are deidentified and located on a secure server in the United States.

## RESULTS

### Patient Characteristics

**Figure 1.**
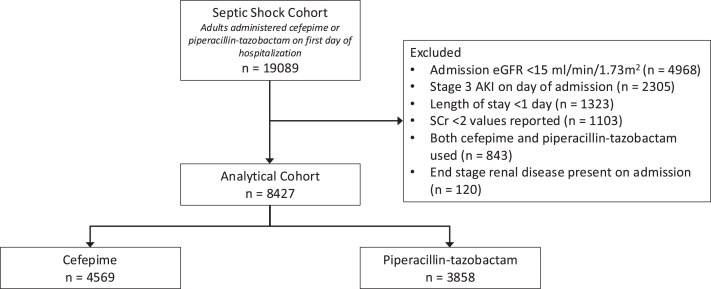
Flow diagram of cohort selection. Abbreviations: AKI, acute kidney injury; CEF, cefepime; eGFR, estimated glomerular filtration rate; PTZ, piperacillin-tazobactam; SCr, serum creatinine.

Of 19 089 adult patients with septic shock who received cefepime or piperacillin-tazobactam, 8427 were included in the final cohort. The flow diagram with reasons for exclusion is provided in Figure 1. Of the final cohort, 4569 received cefepime and 3858 received piperacillin-tazobactam. Patients had a mean (SD) age of 66.2 years (15.2), and 45.3% were female. The mean (SD) APACHE II score was 22 (8), the median CCI was 2 (IQR, 1–3), 23.7% had kidney disease, and 5.0% had moderate to severe liver disease before admission. On the day of admission, the mean (SD) eGFR was 48 (24) mL/min/1.73 m^2^, and 35.0% received invasive mechanical ventilation. Besides cefepime and piperacillin-tazobactam, patients also received vancomycin (79.3%) or other broad-spectrum gram-negative antibiotics (29.4%) before the primary outcome.

The groups were well balanced with regard to all baseline variables (SMD, <0.1) after adjustment (Table 1). The distribution and overlap of propensity scores and plot of SMD for each variable before and after adjustment are provided in Supplementary Figures 1 and 2. Cefepime was used for a median (IQR) of 4 (2–6) days, and piperacillin-tazobactam was used for 4 (2–7 days). A plot of cefepime and piperacillin-tazobactam use during hospitalization is provided in Supplementary Figure 3. In the subset of patients with vancomycin coadministration before the primary outcome, the median duration of vancomycin (IQR) was 3 (2–5) days in both groups (Supplementary Figure 4).

**Table 1.** Baseline Patient Characteristics

Characteristic	Patients, No. (%)^a^			
	Without Adjustment		With Adjustment^b^	
	CEF (n = 4569)	PTZ (n = 3858)	CEF (n = 4593)	PTZ (n = 3850)
Age, mean (SD), y	67.0 (14.8)	65.2 (15.5)	66.2 (15.2)	66.1 (15.2)
Sex				
Female	2124 (45.5)	1691 (43.8)	2072 (54.9)	1743 (54.7)
Male	2445 (53.5)	2167 (56.2)	2521 (45.1)	2107 (45.3)
Race or ethnicity		…		
White	3299 (72.2)	2611 (67.7)	3240 (70.5)	2713 (70.5)
Black	639 (14.0)	604 (15.7)	662 (14.4)	558 (14.5)
Hispanic	386 (8.4)	363 (9.4)	398 (8.7)	332 (8.6)
Asian	81 (1.8)	100 (2.6)	91 (2.0)	83 (2.2)
Other	112 (2.5)	146 (3.8)	156 (3.4)	125 (3.2)
Unknown	52 (1.1)	34 (0.9)	46 (1.0)	39 (1.0)
BMI, mean (SD)^c^	27.9 (7.3)	27.3 (7.6)	28.0 (7.3)	27.9 (7.6)
APACHE II score, mean (SD)	23 (8)	22 (7)	23 (8)	23 (7)
CCI, median (IQR)	2 (1–3)	2 (1–3)	2 (1–3)	2 (1–3)
Liver disease	206 (4.5)	218 (5.7)	232 (5.1)	199 (5.2)
Renal disease	1101 (24.1)	894 (23.2)	1116 (24.3)	917 (23.8)
Laboratory values, mean (SD)				
eGFR, mL/min	47 (22)	50 (26)	48 (23)	48 (23)
Creatinine, mg/dL^d^	1.6 (0.7)	1.6 (0.7)	1.6 (0.7)	1.6 (0.7)
Lactate, mmol/L^d^	4.3 (3.2)	4.5 (3.4)	4.3 (3.2)	4.4 (3.3)
Chloride, mEq/L^d^	103 (8)	103 (8)	103 (8)	103 (8)
Invasive ventilation	1590 (34.8)	1362 (35.3)	1617 (35.2)	1339 (34.8)
Antibiotics^e^				
Vancomycin	3833 (83.9)	2847 (73.8)	3670 (79.9)	3065 (79.6)
Gram-negative broad-spectrum^f^	1362 (29.8)	1117 (29.0)	1354 (29.5)	1151 (30.0)
Infection source^g^				
Central nervous system	41 (0.9)	28 (0.7)	37 (0.8)	32 (0.8)
Lung	1824 (39.9)	1447 (37.5)	1806 (39.3)	1500 (39.0)
Intra-abdominal	528 (11.6)	799 (20.7)	736 (16.0)	610 (15.9)
Skin and soft tissue	406 (8.9)	360 (9.3)	418 (9.1)	344 (8.9)
Genitourinary	1776 (38.9)	1356 (35.1)	1714 (37.3)	1440 (37.4)
Teaching hospital	2009 (44.0)	1839 (47.7)	2062 (44.9)	1742 (45.2)
Hospital area				
Rural	528 (11.6)	812 (21.0)	787 (17.1)	621 (16.1)
Urban	4041 (88.4)	3046 (79.0)	3806 (82.9)	3229 (83.9)
Hospital region				
Midwest	437 (9.6)	1011 (26.2)	811 (17.7)	665 (17.3)
Northeast	394 (8.6)	170 (4.4)	306 (6.7)	261 (6.8)
South	3665 (80.2)	2490 (64.5)	3331 (72.5)	2805 (72.9)
West	73 (1.6)	187 (4.8)	145 (3.2)	119 (3.1)

Abbreviations: APACHE, Acute Physiology and Chronic Health Evaluation; BMI, body mass index; CCI, Charlson Comorbidity Index; CEF, cefepime; eGFR, estimated glomerular filtration rate; IQR, interquartile range; PTZ, piperacillin-tazobactam; SD, standard deviation.

^a^Data represent no. (%) of patients unless otherwise specified.

^b^All standardized mean differences after adjustment were <0.1.

^c^BMI calculated as weight in kilograms divided by height in meters squared.

^d^Serum concentrations.

^e^Use before primary outcome.

^f^Includes aminoglycosides, carbapenems, ceftazidime, ceftazidime-avibactam, cefidericol, ceftolozane-tazobactam, imipenem-relebactam, meropenem-vaboractam, or tigecycline.

^g^May not add to 100% as not mutually exclusive.

### Outcomes

In the IPTW cohort, stage 3 AKI occurred in 9.9% with cefepime and 9.8% with piperacillin-tazobactam (OR, 0.98 [95% CI, .84–1.15]; *P* = .82). In terms of secondary outcomes, there were no significant differences between cefepime and piperacillin-tazobactam with regard to RRT, in-hospital death, MAKEs, stage 1 or 2 AKI, maximum recorded SCr value, or hospital length of stay (Table 2).

**Table 2.** Patient Outcomes

Outcomes	Patients, No. (%)^a^		OR/Coefficient^b^ (95% CI)	*P* Value
	CEF (n = 4593)	PTZ (n = 3850)		
Primary				
Stage 3 AKI^c^	456 (9.9)	376 (9.8)	0.98 (.84–1.15)	.82
Secondary				
RRT	112 (2.4)	69 (1.8)	0.73 (.53–1.01)	.055
Death	1036 (22.5)	819 (21.3)	0.93 (.83–1.04)	.20
MAKE^d^	1160 (25.2)	914 (23.8)	0.92 (.83–1.03)	.14
Stage 1 AKI^c^	1354 (29.5)	1159 (30.1)	1.03 (.93–1.14)	.56
Stage 2 AKI^c^	1386 (30.2)	1149 (29.9)	0.99 (.89–1.09)	.77
Maximum SCr, median (IQR), mg/dL	1.7 (1.1–2.3)	1.7 (1.1–2.3)	0.03^e^ (−.14 to .19)	.76
Hospital LOS, median (IQR), d	8 (5–14)	9 (5–15)	0.45^e^ (−.04 to .95)	.07

Abbreviations: AKI, acute kidney injury; CEF, cefepime; CI, confidence interval; IQR, interquartile range; LOS, length of stay; MAKE, major adverse kidney event; OR, odds ratio; PTZ, piperacillin-tazobactam; RRT, renal replacement therapy; SCr, serum creatinine.

^a^Data represent no. (%) of patients unless otherwise specified.

^b^Data represent OR unless identified as coefficient.

^c^AKI stage defined according to Kidney Disease: Improving Global Outcomes (KDIGO) definition.

^d^MAKE defined as stage 3 AKI at discharge or RRT (or other dialysis) or death

^e^Coefficient.

**Figure 2.**
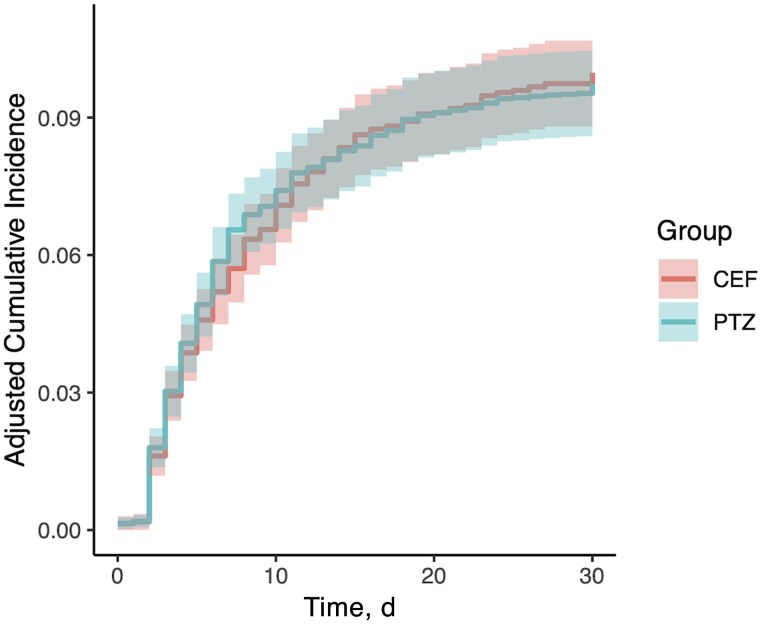
Adjusted cumulative incidence of stage 3 acute kidney injury, with curve adjusted for baseline covariates and with death as a competing risk. Abbreviations: CEF, cefepime; PTZ, piperacillin-tazobactam.

**Figure 3.**
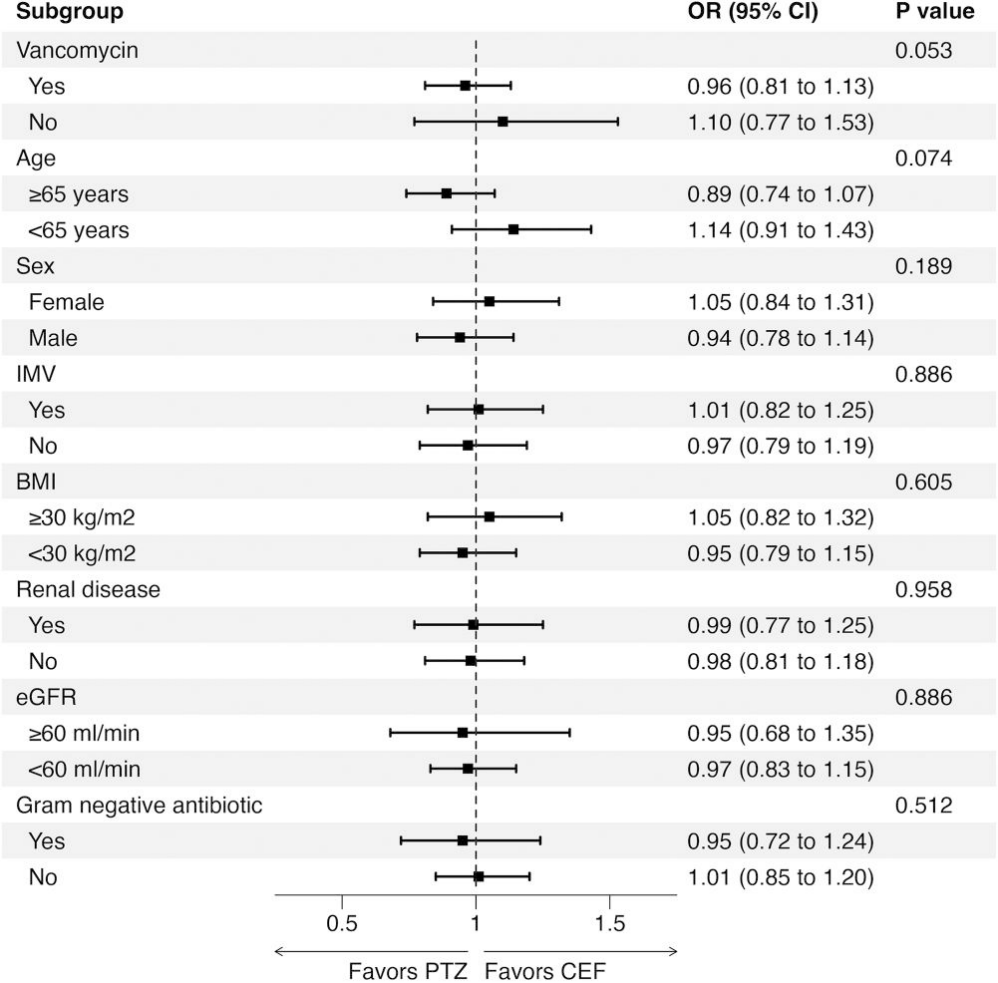
Subgroup analyses for the primary outcome of stage 3 acute kidney injury. Parameters were measured at baseline, and vancomycin and gram-negative antibiotics are at any time before the primary outcome measure. *P* values were obtained using an interaction test to identify heterogeneity and are not adjusted for multiplicity. Abbreviations: BMI, body mass index (calculated as weight in kilograms divided by height in meters squared); CEF, cefepime; CI, confidence interval; eGFR, estimated glomerular filtration rate; IMV, invasive mechanical ventilation; OR, odds ratio; PTZ, piperacillin-tazobactam.

The first sensitivity analysis, using a mixed effects model with hospital as a random effect, supported the primary analysis (OR, 0.98 [95% CI, .84–1.14]; *P* = .76). In the second sensitivity analysis, with death as a competing risk, there was no significant difference between groups in terms of stage 3 AKI (subdistribution hazard ratio, 0.96 [95% CI, .82–1.11]; *P* = .55). The adjusted cumulative incidence plot is provided in Figure 2. The third sensitivity analysis, comparing groups with ≥2 days of index antibiotic use (days 1 and 2), also showed no significant difference between groups (OR, 0.91 [95% CI, .77–1.07]; *P* = .26). In the subgroup analyses, there were no significant associations within any of the predefined subgroups (Figure 3). There was no significant difference between groups whether vancomycin was coadministered (OR, 0.96 [95% CI, .81–1.13]; *P* = .58) or not (1.10 [.77–1.53]; *P* = .56).

## DISCUSSION

The key finding of this study is that in patient with septic shock, there was no difference in the occurrence of AKI between cefepime and piperacillin-tazobactam. The study is consistent with findings from the ACORN (Antibiotic Choice on Renal Outcomes) trial and extends it to the septic shock population, whose members are particularly at risk for AKI. We also did not find any heterogeneity of effect in any of the predefined subgroups. This included patients with or without concurrent vancomycin use.

The ACORN trial randomized to cefepime or piperacillin-tazobactam patients (n = 2634) who presented with infection to the emergency department or medical intensive care unit (ICU) [2]. The primary outcome was the highest stage of AKI, based on a 5-level ordinal scale ranging from no AKI to death within 14 days. There was no significant difference between the groups (OR, 0.95, [95% CI, .80–1.13]; *P* = .56) with regard to the primary outcome. Stage 3 AKI occurred in 7.0% of patients with cefepime and 7.5% with piperacillin-tazobactam. The incidence of stage 3 AKI in our study was slightly higher in both groups, which can be attributed to the more severely ill population. In the ACORN trial only 4.2%–6.5% of patients were in the ICU at the time of enrollment, and close to 90% of those enrolled from the emergency department, subsequently went to a non-ICU ward. Only 13% of the cohort overall had septic shock. Similar to our study, 77%–78% of patients in ACORN received vancomycin. There was no heterogeneity of effect based on vancomycin use, which is consistent with the ACORN trial.

A recent observational study (n = 7569) has compared cefepime and piperacillin-tazobactam with regard to the 90-day mortality rate in adult patients with sepsis who are also treated with vancomycin [13]. The study excluded patients with indications for antianaerobic therapy. Piperacillin-tazobactam was associated with a higher 90-day mortality rate than cefepime (22.5% vs 17.%, respectively; *P* = .002). The study did not evaluate AKI as an outcome. However, it generates the hypothesis that anaerobic depletion with use of piperacillin-tazobactam may worsen clinical outcomes. Interestingly, a post hoc analysis of this study with 14-day outcomes similar to those of ACORN showed no significant difference in mortality rates between groups. The duration of our assessment of mortality rates is more similar to that in the ACORN trial, as we did not have data after hospitalization. Moreover, our primary focus was on AKI and the relevant time period of this outcome is during the index hospitalization.

Before the ACORN trial, meta-analyses have shown that the combination of piperacillin-tazobactam and vancomycin increases the risk of AKI compared with the use of vancomycin alone [13–15]. This risk is not mitigated by using area under the curve–based vancomycin dosing [16]. Furthermore, the addition of a β-lactam to vancomycin is often necessary for the management of sepsis. A meta-analysis in patients in the ICU compared piperacillin-tazobactam plus vancomycin versus alternative β-lactams plus vancomycin. Cefepime was the most common alternative β-lactam. The meta-analysis of 9 studies including cefepime showed higher rates of AKI with piperacillin-tazobactam plus vancomycin than with cefepime plus vancomycin (OR, 1.70, [95% CI, 1.36–2.12]; *I*^2^ = 83%; *P* < .001) [14]. All stages of AKI were pooled together in this analysis. The studies had a high risk of bias, substantial heterogeneity, differences in definitions of AKI, and relatively small sample sizes, and all were retrospective. Using more robust methods, the findings from our investigation are more consistent with ACORN than these observational studies.

KDIGO definitions for AKI currently incorporate SCr, which may not be the ideal biomarker for identifying AKI. In one observational study, piperacillin-tazobactam plus vancomycin was associated with a higher incidence of SCr-defined AKI than cefepime plus vancomycin (rate ratio, 1.34 [95% CI, 1.01–1.78]). However, there was no significant association with cystatin C increase ≥50% (rate ratio, 0.95 [95% CI, .44–2.02]), a kidney biomarker that is not affected by tubular secretion. This finding suggests that the AKI observed in previous studies using SCr-defined AKI may represent pseudonephrotoxicity.

Although this is one of the largest observational studies on this topic, there are a few limitations to consider. First, the definition of AKI is dependent on baseline SCr, and we had to make assumptions for this as recommended by KDIGO, similarly to other major clinical trials, such as ACORN. We also included more patient-centered measures such as RRT, in-hospital death, and MAKEs as secondary outcomes. None of these differed between groups. Second, there is potential for unmeasured confounding, as with any observational study. We used robust approaches to minimize confounding and obtain well-balanced groups, but residual confounding may remain. In addition, 3 sensitivity analyses were conducted, and all supported the main analysis. Third, there were missing data in some baseline parameters, although we used multiple imputation, the least biased approach to manage missing data [17]. Fourth, the short duration of use of the index antibiotics, with or without vancomycin coadministration, may suggest that the study was not poised to evaluate this interaction.

In conclusion, in this large retrospective, multicenter cohort study conducted in 220 geographically diverse community and teaching hospitals across the United States, the proportion of adult patients with septic shock on hospital admission who experienced stage 3 AKI did not differ significantly between those receiving cefepime and those receiving piperacillin-tazobactam. Similarly, there were no significant differences between cefepime and piperacillin-tazobactam for any of the predefined subgroups, including concomitant vancomycin therapy, or for the secondary outcomes of RRT, in-hospital death, MAKEs, stage 1 or 2 AKI, maximum recorded SCr level, or hospital length of stay.

## Supplementary Material

ciae600_Supplementary_Data

## Supplementary Data

Supplementary materials are available at *Clinical Infectious Diseases* online. Consisting of data provided by the authors to benefit the reader, the posted materials are not copyedited and are the sole responsibility of the authors, so questions or comments should be addressed to the corresponding author.

## Notes

***Author Contributions***. Conceptualization and writing—original draft: A .E. P., D. E. N., and B. L. E. Data acquisition: A. E. P. and B. L. E. Formal analysis: A. E. P. Methodology and writing—review and editing: All authors.

***Data availability.*** Due to contractual agreements for the PINC AI Healthcare Database, the data are not publicly available

***Financial support***. This work was supported by the University of Arizona and the University of Sydney (internal institutional funds).
