# Supplementary material for: A National Retrospective Cohort Study Comparing the Effects of Cefepime Versus Piperacillin-Tazobactam on the Development of Severe Acute Kidney Injury in Patients With Septic Shock
Source: Clin Infect Dis. 2024 Dec 5;80(4):770–6. doi: 10.1093/cid/ciae600 (PMC12043064; doi:10.1093/cid/ciae600)
Supplement: ciae600_Supplementary_Data [file ciae600_supplementary_data.docx]

***Comparison of cefepime and piperacillin-tazobactam on the development of severe acute kidney injury in patients with septic shock***

Supplementary Appendix

Table of Contents

[eTable 1. Kidney Disease: Improving Global Outcomes (KDIGO) definitions 2](#_Toc182832247)

[eFigure 1. Distribution of propensity score before and after weighting 3](#_Toc182832248)

[eFigure 2. Plot of standardized mean differences 4](#_Toc182832249)

[eFigure 3. Plot of cefepime and piperacillin use per day 5](#_Toc182832250)

[eFigure 4. Plot of the vancomycin exposure 6](#_Toc182832251)

# eTable 1. Kidney Disease: Improving Global Outcomes (KDIGO) definitions

| **Stage** | **Serum creatinine†** |
| --- | --- |
| 1 | 1.5-1.9 times baseline OR ≥0.3 mg/dl increase |
| 2 | 2.0-2.9 times baseline |
| 3 | 3.0 times baseline OR increase to ≥4.0 mg/dl OR initiation of renal replacement therapy |

†Only serum creatinine component used as urine output was not available

# eFigure 1. Distribution of propensity score before and after weighting


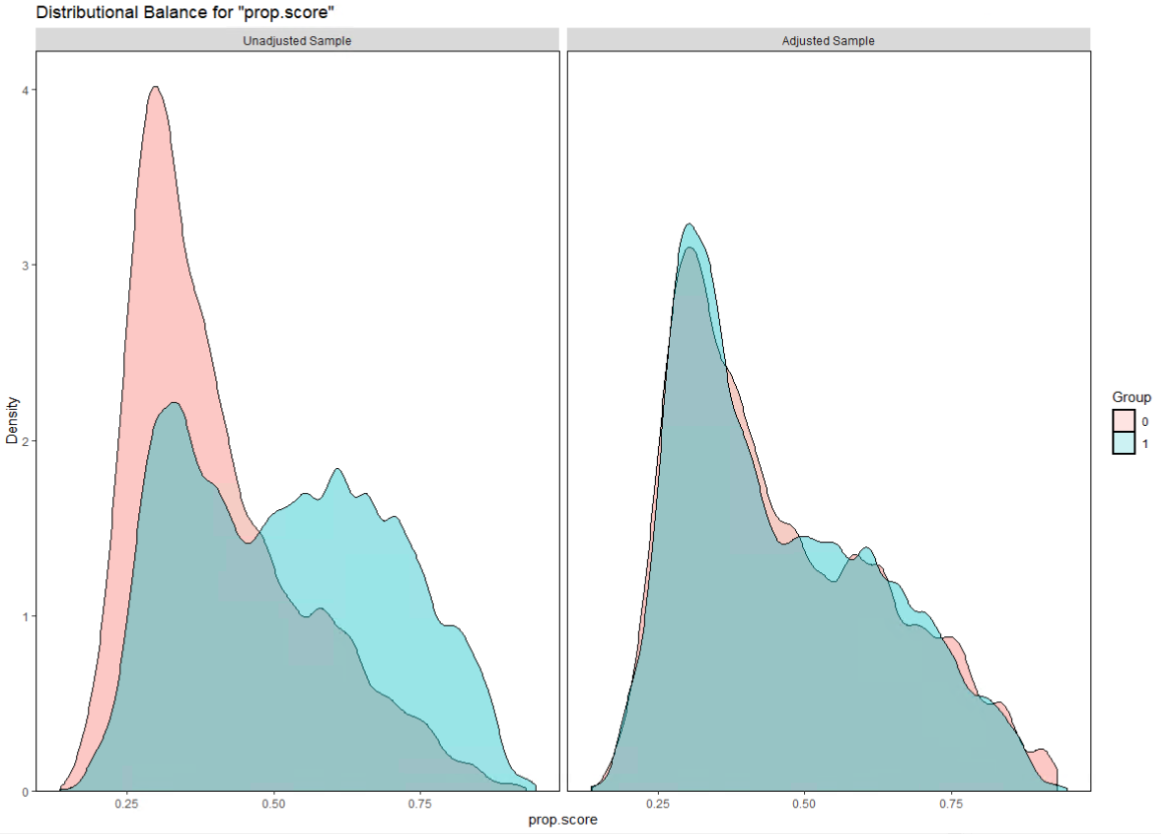


0 = Cefepime

1 = Piperacillin-tazobactam

# eFigure 2. Plot of standardized mean differences


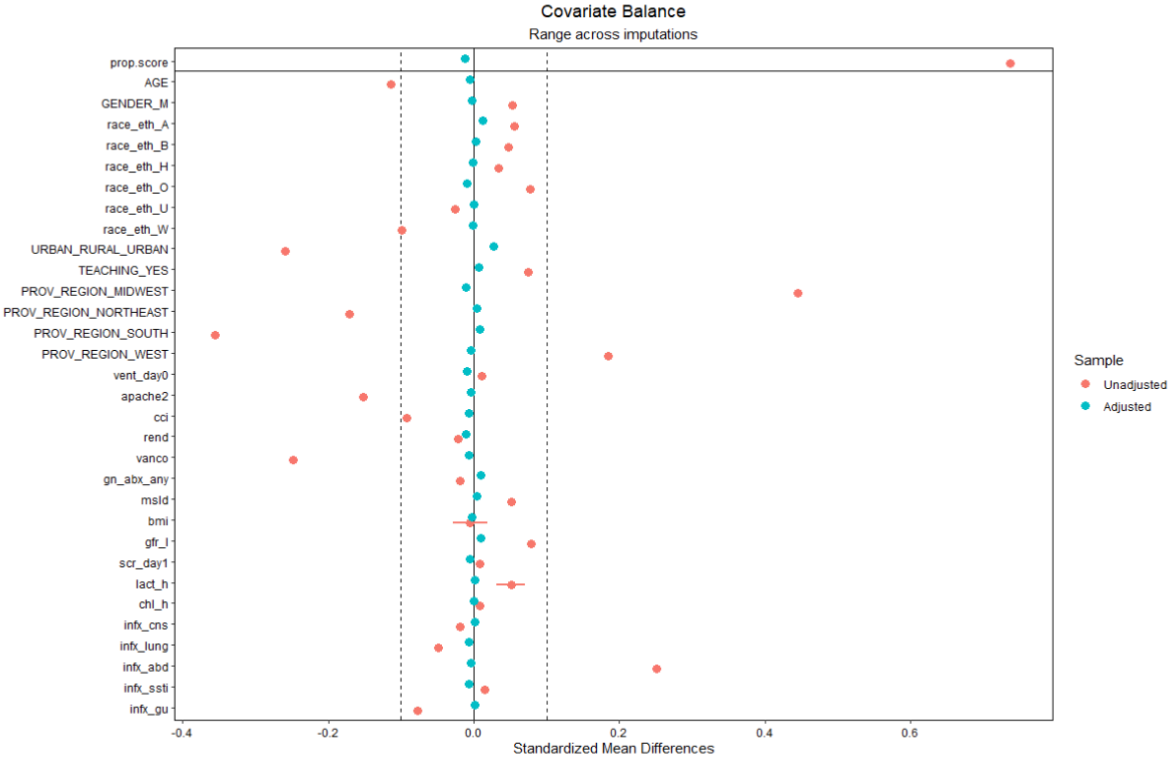


# eFigure 3. Plot of cefepime and piperacillin use per day


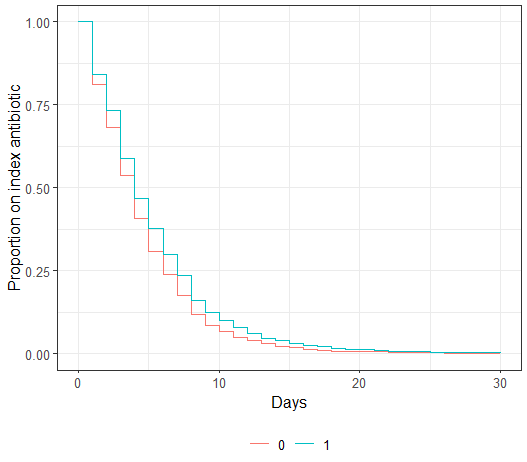


0 = Cefepime

1 = Piperacillin-tazobactam

# eFigure 4. Plot of the vancomycin exposure

**
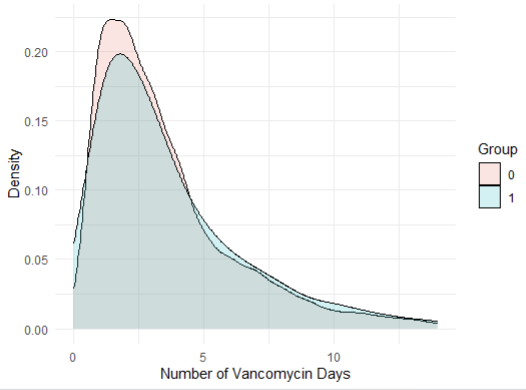
**

0 = Cefepime

1 = Piperacillin-tazobactam

Distributional plot of the duration of vancomycin in subset exposed to vancomycin prior to the primary outcome
